# Malignant melanoma in Chile: different site distribution between private and state patients

**DOI:** 10.1186/0717-6287-47-34

**Published:** 2014-07-24

**Authors:** Viviana B Zemelman, Carlos Y Valenzuela, Ivo Sazunic, Irene Araya

**Affiliations:** Department of Dermatology, Faculty of Medicine, University of Chile, Santiago, Chile; Human Genetics Program, Faculty of Medicine, University of Chile, Santiago, Chile; Holanda 3444, Dpto 32, Nuñoa, Santiago, Chile

**Keywords:** Chile, Malignant melanoma, Site distribution, Socioeconomic strata

## Abstract

**Background:**

The body site location of primary Malignant Melanoma (MM) has been correlated with prognosis and survival. Ethnic, genetics, sun exposure factors are related to the anatomical distribution of MM. Low and high socioeconomic strata in Chile differ in ethnic, genetic and cultural conditions. The purpose of this study was to analyze the anatomical MM distribution in the Chilean population in both strata searching for differences due to their ethno-genetic-cultural differences. Records of 1148 MM, 575cases from state hospitals (Low Socioeconomic Strata, LSS) and 573 cases from private clinics (High Socioeconomic Strata, HSS) were analyzed by body site.

**Results:**

Females from LSS showed a higher number of MM in soles, cheeks, and around the eye area. Females from the HSS showed a higher number of MM in dorsal feet and dorsal hands. Males from LSS showed a higher number of MM in soles, around the eye area, and cheeks. However, males from HSS showed a higher number of MM in the trunk, and in the arms. Acral MM was significantly higher in LSS than in the HSS in both sexes. The Chilean population from the HSS and LSS showed differences in the distribution of MM by site. Furthermore, gender differences in the proportion of MM analyzed by anatomical site are observed in both strata.

**Conclusions:**

Results show evidence that differential genetics factors, sun exposure, or other environmental or cultural factors of both strata may account for these differences.

## Background

The incidence and mortality of Malignant Melanoma (MM) have increased many folds over the past several decades in the Caucasian population and in Chile [1–3]. Anatomic location of MM differs according to the race and gender of the patient. MM is mainly located in the trunk in Caucasian males and is mainly located on the legs in females. Furthermore, in Asian and Negroid patients, MM is mainly located on the palms and soles [4]. The anatomical location of primary cutaneous MM has been correlated with prognosis and survival. High-risk sites of MM in stage I include the scalp, trunk, hands, feet, genital area [5]. On the other hand, patients with MM located on the soles show poor prognosis and poor survival (five year survival rate of 35%) [6]. The effect of primary site on prognosis has been further analyzed by Gillgren et al. [7].

The Chilean population descends primarily from the union between Caucasian men (mostly Spaniards) and Amerindian women. At the present time, the Chilean population mixture can be described as follows: the high socio-economic stratum (5% of the population) with 5% of Amerindian mixture; the middle stratum (20% of population) with 20% of Amerindian mixture and the low stratum (75% of population) with 35 to 40% of Amerindian mixture [8–10]. In Chile, people who cannot pay their health attention and people with the lowest income use the public healthcare system. People that can pay for their medical attention use the private healthcare systems. Thus, people seen in public healthcare services belong to the middle - low and low socioeconomic strata and people seen in private services belong to middle-high and high strata; people from the high socioeconomic strata do not use the public service and the low strata rarely use the private healthcare system. Moreover, the insurance policy conditions of both systems that are based mostly on income tend to maintain these socioeconomic differences [11].

But the most important difference, for our present study, is the ethno-genetic clear difference of socioeconomic strata; persons from the public health service have near 40% of Amerindian mixture, while persons from the private service have at most 20% of Amerindian mixture [9]. The population going to the private health system in Chile is mainly Caucasian. It is known that Amerindians are descended from Asian people and Asian people differ greatly from Caucasians in the location of MM. Asians have more acral MM than Caucasians [12]. A previous study on the skin colour in the Chilean population showed that individuals from the low stratum were darker than those from the high stratum [13]. These socioeconomic and ethno-genetic conditions of the Chilean society lead us to think that acral MM will be more frequent in the population which uses the State Health Service. Recent research on MM points to the coexistence of several biological pathways linked to the anatomical site of lesion, which could lead to this neoplasm [14]. Because prognosis and survival of MM has been related to anatomical location we considered it important to analyze the anatomical MM distribution in the Chilean population seen in public (mostly low socioeconomic strata, [15]) and private (mostly high socioeconomic strata, this study) healthcare services due to the different ethnic composition of populations of both strata.

## Results and discussion

Table 1 shows the anatomical site of 575 MM (360 MM in females and 215 MM in males) from state hospitals (LSS) and 573 MM (329 MM in females and 244 MM in males) from private clinics (HSS). Only data from LSS included Non-Specified anatomical location. Significant differences by specific location between both strata are indicated in italics.

**Table 1:** **Anatomical site by gender and by health system**

	Public health system (LSS)					Private health system (HSS)				
Anatomical site	Female		v Male		Total	Female		Male		Total
	n	%	n	%	n	n	%	n	%	n
Forehead	8	2.2	3	1.4	11	6	1.8	7	2.9	13
Cheeks	*63*	*17.4*	*17*	*7.9*	*80*	*23*	*7.0*	*9*	*3.7*	*32*
Middle face	10	2.8	4	1.9	14	11	3.3	14	5.7	25
Chin	1	0.3	2	0.9	3	2	0.6	0	0.0	2
Ears	3	0.8	9	4.2	12	3	0.9	8	3.3	11
Scalp	2	0.6	5	2.3	7	1	0.3	7	2.9	8
Eye area	*11*	*3.1*	*8*	*3.7*	*19*	*3*	*0.9*	*1*	*0.4*	*4*
Face/NS	9	2.5	3	1.4	12	0	0.0	0	0.0	0
Neck	3	0.8	3	1.4	6	3	0.9	7	2.9	10
Trunk	*37*	*10.2*	*31*	*14.4*	*68*	*57*	*17.4*	*76*	*31.1*	*133*
Abdomen	1	0.3	3	1.4	4	4	1.2	5	2.0	9
Genital area	5	1.4	0	0.0	5	6	1.8	0	0.0	6
Buttock	2	0.6	1	0.5	3	1	0.3	0	0.0	1
Legs	73	20.2	21	9.8	94	90	27.5	28	11.5	118
Arms	*25*	*6.9*	*6*	*2.8*	*31*	*40*	*12.2*	*23*	*9.4*	*63*
Palms	1	0.3	1	0.5	2	1	0.3	0	0.0	1
Dorsal hands	*0*	*0.0*	3	1.4	3	*5*	*1.5*	2	0.8	7
Hands, NS	11	3.1	6	2.8	17	0	0.0	0	0.0	0
Soles	*28*	*7.8*	*27*	*12.6*	*55*	*3*	*0.9*	*6*	*2.5*	*9*
Foot, NS	20	5.6	19	8.8	39	0	0.0	0	0.0	0
Dorsal foot	*0*	*0.0*	3	1.4	3	*9*	*2.7*	3	1.2	12
Body NS	6	1.7	5	2.3	11	0	0.0	0	0.0	0
Location NS	41	11.4	35	16.2	76	61	18.5	48	19.7	109
Total	360	100.0	215	100.0	575	329	100.0	244	100.0	573

### Comparison between both strata

Both strata (females and males) were highly different in the anatomical distribution of MM (χ^2^_8_ = 105.4; P < 1.2×10^−7^). These results were obtained from the nine most frequent anatomical sites excluding the non-specified site (NS). A higher number of MM in the LSS was observed in soles and eye area. However, a higher number of MM in the HSS was observed on the arms and on the trunk. Females from LSS showed a higher number of MM in soles, cheeks, and in eye area. Furthermore, females from the HSS strata showed a higher number of MM in dorsal feet and dorsal hands. Comparing males from both strata, males from LSS showed a higher number of MM in soles, eye area, and cheeks. However, males from HSS showed a higher number of MM in trunk and arms, but no significant differences were found in dorsal hands and dorsal feet. Acral MM (palms and soles) in females and males from LSS were 8.1% and 13.1%, respectively, however, in HSS were 1.2% and 2.5%, respectively; a highly significant difference between both strata.

The structure of body site distribution of MM in both strata is clearly different; the difference found in the acral MM is remarkable. The higher proportion of acral MM in females and males from the low socioeconomic strata indicates a genetic factor. In Chile, the ethnic composition of the HSS includes: 5% of Amerindian mixture and 95% Caucasian, while the LSS has around 40% of Amerindian mixture and 60% Caucasian [9]. The highest frequency of Acral MM has been described in the Black and Asian populations; as we know Amerindians descend from Asian populations. The acral MM found in our investigation was mainly located on the soles. Soles are the main location of MM in the Asian and African populations [16, 17]. Other differences were found: we observed a higher frequency of MM in the trunk of the HSS. The presence of nevi has been showed to be associated to race. Caucasians would have more nevi than non Caucasians [18]. Furthermore, the association of MM–nevus has been described, being stronger on the trunk than in other anatomical locations. [14]. Also, a significantly higher proportion of MM in cheeks and in the eye area in both sexes from low socioeconomic strata may be explained by the higher chronic sun exposure of this population; often the MM of the head and neck are associated to this pattern of sun exposure. Acral MM aetiological factors differ from those MM at other sites. Ultraviolet (UV) radiation probably plays an insignificant role in unexposed sites like palms and soles. Furthermore, the particular aggressiveness of the acral lentiginous melanoma subtype, histological type of MM characterized by its predilection for palms and soles has been described. Also, molecular studies have shown a higher frequency of c-kit mutations associated with these tumors [19].

### Comparisons between females and males between and within the strata

In LSS, females showed a significantly higher number of MM on the cheeks, legs, arms, and genital area than males. However, males showed a significantly higher number of MM on the ears, dorsal hands; soles and feet (see [15]). In the HSS, females showed a significantly higher number of MM on the legs and genital area than males. However, males showed a significantly higher number of MM on the ears, trunk and scalp.

MM arising in the genital area is rare, we had only a few cases, but our results confirm the findings in other population that show a higher frequency of MM in the genital area in females than males [15, 20]. On the other hand, oncogenic human papillomavirus (HPV) types such as HPV 16, has been detected in two cases of vulvar MM. [21]. The aetiology of genital MM remains unknown, and the presence of virus HPV has not been correlated with the development of MM. The role of oncogenic virus in the development of genital MM is a controversial subject. Besides of the differences in genital MM, females from both strata show a higher number of MM in the legs than males; these results are in agreement with those obtained by others authors and are probably due to many different sexual dimorphic cultural or genetic factors [13, 15]. Moreover, males from both strata showed a higher number of MM in ears than females probably due to sun protection by hair, in agreement with results of other authors [22]. The fact that females from HSS showed more MM in dorsal feet and in dorsal hands than females from LSS is not clear for us; it may be due to different factors, as for example skin type and sun exposure.

The comparison between females and males from the low socioeconomic strata has already been analysed [15]. Males from high socioeconomic strata had higher proportions of MM in trunk, scalp and ears than females. This higher proportion of MM in ears in males agrees with that found in the low socioeconomic strata, this fact may be explained by the higher sun exposure of ears in males than in females. The higher proportion of MM in male scalp is probably due to alopecia (Caucasian); this phenomenon was absent in the Chilean low strata. The fact that males have more proportion of MM on the trunk than females matches the results obtained in Caucasian population where males had a higher risk of developing MM in the trunk [14]; besides that, it has been observed that the presence of nevi and development of MM is different according to the anatomical location. MM from different body sites may have different aetiologies, a recent case control study found that the number of moles was more strongly related to MM of the trunk than that of the head and neck [23]. As we said previously, males have more MM on the trunk than females in the HSS, these results are genetically established and are in agreement with the Caucasian population.

### Average age of patients

#### HSS

The average age of females was 50.3 years old (SD = 15.5). The average age of males was 54.1 (SD = 17.2); the difference between the averages was significant (P = 0.014). The average age of the total was 51.9 (SD = 16.3).

#### LSS

The average age of females was 60.1 years old (SD = 17.1). The average age of males was 60.7 (SD = 16.1). The average age of the total was 60.3 (SD = 16.7); the difference of averages was not significant.

The comparison between the average ages of both strata was highly significant, for males, females, and the total (P < 10^−6^).

The younger age of MM patients from HSS in our investigation may be explained by an early diagnosis of MM due to an easier access to medical and dermatological care due to a better economical situation. Also, patients from HSS are better educated, so they are more aware of skin cancer, thus they recognize signs of MM earlier. The higher prevalence of facial MM in LSS may be partially explained by the older age of the patients from these strata which imply a higher level of chronic sun exposure. However the great differences in body site distribution of MM between both strata, as for example in acral MM can difficultly be due to the difference in age.

### Limitations of the study

This is a retrospective study with data bank files. We cannot access new data of the patients such as income, occupation or life-style. We based our research on the clear socioeconomic and ethno-genetic segregation that the Chilean health systems showed in previous studies [8–11]). The main differences in the MM distribution between both strata found in our study are mainly due to the ethnic differences between both populations. However, we have observed some differences between both strata that are more related to other risk factors of MM such as sun exposure differences or differences in phenotype. Unfortunately, in this study, we could not measure the sun exposure of each individual, but we may assume the differences in sun exposure of the total population of both strata, since we know their different life styles.

## Conclusions

Despite notable limitations mainly due to the fact that this is a retrospective study with data bank files and we cannot access further data on the patients, we have established that the anatomical MM distribution in the Chilean population seen in public (mostly low socioeconomic strata) and private (mostly high socioeconomic strata) healthcare services is different, we showed that acral MM was significantly higher in LSS that in the HSS. This is mainly due to the different ethnic composition of populations of both strata; population from the LSS in Chile is 40% Amerindian and 60% Caucasian. Furthermore, our main findings showed that males from HSS showed a higher percentage of MM on the trunk. These results are in agreement with those found in the Caucasian population. These results support the idea that MM location is, in some way, associated with the ethnic composition of the studied population. However, studies examining other melanomas risk factors should be taken into account in future studies.

## Methods

### Chilean health system and socioeconomic strata

The Chilean health system has two sectors, public and private. The public sector covers the rural and urban poor population (low, middle-low socioeconomic strata with around 40% of Amerindian mixture). The private sector covers mostly the upper, middle-upper class and the high income population (high and middle-high socioeconomic strata, with at most 20% of Amerindian mixture) [8–11].

We analysed all histopathological reports (600,000) in the Histopathology Department from five major state hospitals of Santiago between 1992 and 2001, belonging to the Chilean State Healthcare Service (low socioeconomic strata, LSS, [15]). These hospitals are used by a population of approximately 2 million people, approximately one third of the total population of Santiago. All samples with histopathological diagnosis of MM were included in the study. We studied 575 primary MM (360 females, 215 males) from the State System. Also, all histopathological reports (1994–2008) from a private health centre (high socioecomic strata, HSE) were analyzed. A total of 573 primary MM (329 females and 244 MM males) from private patients were included. From both systems 1,148 MM were analyzed according to the anatomical location and gender. Patients with metastatic and recurrent MM were excluded from the study.

### Statistical analysis

The comparison of the distribution between both populations was performed by a chi-square test, with a minimal significance level of 0.05; the degrees of freedom were subscripted. The Poisson distribution was used for numbers lower than six. The difference in the average age was tested by a Student t test.

## References

[1] Arnold M, Holterhues C, Hollestein LM, Coebergh JW, Nijsten T, Pukkala E, Holleczek B, Tryggvadóttir L, Comber H, Bento MJ, Diba CS, Micallef R, Primic-Žakelj M, Izarzugaza MI, Perucha J, Marcos-Gragera R, Galceran J, Ardanaz E, Schaffar R, Pring A, de Vries E (2013). Trends in incidence and predictions of cutaneous Melanoma across Europe up to 2015. J Eur Acad Dermatol Venerol.

[2] Zemelman V, Roa J, Díaz C, Araya I, Zamalloa G, Faundez E (2001). Aumento de la incidencia del cáncer cutáneo en hospitales públicos de la Región Metropolitana (1992–1998). Rev Chil Dermatol.

[3] Zemelman V, Garmendia ML, Kirschbaum A (2002). Malignant Melanoma Mortality in Chile (1988–98). Int J Dermatol.

[4] Grichnik JM, Rhodes AR, Sober AJ, Wolff K, Goldsmith LA, Katz SI, Gilchrest BA, Paller AS, Leffell DJ (2008). Melanocytic tumors. Fitzpatrick’s Dermatology in General Medicine.

[5] Rogers GS, Kopf AW, Rigel DS, Friedman RJ, Levine JL, Levenstein M, Bart RS, Mintzis MM (1983). Effect of anatomical location on prognosis in patients with clinical stage I melanoma. Arch Dermatol.

[6] Seiji M, Takematsu H, Hosokawa M, Obata M, Tomita Y, Kato T, Takahashi M, Mihm MC (1983). Acral melanoma in Japan. J Invest Dermatol.

[7] Gillgren P, Brattstrőm G, Frisell J, Persson JO, Ringborg U, Hansson J (2005). Effect of primary site on prognosis in patients with cutaneous malignant melanoma. A study using a new model to analyse anatomical locations. Melanoma Res.

[8] Valenzuela CY (1984). Sociogenetic reference limits for public health studies in Chile. Rev Chil Pediatr.

[9] Valenzuela CY (2011). Human Sociogenetics. Biol Res.

[10] Valenzuela CY, Acuña MP, Harb Z (1987). Sociogenetic gradient in the Chilean population. Rev Med Chile.

[11] Becerril-Montekio V, Reyes JDED, Manuel A (2011). The health system of Chile. Salud Publica Mex.

[12] Lee HY, Chay WY, Tang MB, Chio MT, Tan SH (2012). Melanoma: differences between Asian and Caucasian patients. Ann Acad Med Singapore.

[13] Zemelman V, Von Beck P, Alvarado O, Valenzuela CY (2002). Sexual dimorphism in skin, eye and hair color and the presence of freckles in Chilean teenagers from two socio-economic strata. Rev Med Chile.

[14] Cho E, Rosner BA, Colditz GA (2005). Risk factors for melanoma by body site. Cancer Epidemiol Biomarkers Prev.

[15] Zemelman V, Roa J, Ruiz Tagle S, Valenzuela CY (2006). Malignant Melanoma in Chile: an unusual distribution of primary sites in men from low socioeconomic strata. Clin Exp Dermatol.

[16] Elwood JM, Gallagher RP (1983). Site distribution of malignant melanoma. Can Med Assoc J.

[17] Brisson C, Reynaud-Hautin C, Bure E, Chatal M, Hadet-Riegert M, Rafstedt P, Humeau S, Kerangueven J, Leroux E, Legroux J, Martin MP, Nogues M, Remond L, Ottavy N, Thebaud Y, Patarin M, Clement A, Proux M (2003). Prospective epidemiological study on cutaneous melanomas in the Vendee area. Ann Dermatol Venereol.

[18] Rampen FH, De Wit PE (1989). Racial differences in mole proneness. Acta Derm Venereol.

[19] Beadling C, Jacobson-Dunlop E, Hodi FS, Le C, Warrick A, Patterson J, Town A, Harlow A, Cruz F, Azar S, Rubin BP, Muller S, West R, Heinrich MC, Corless CL (2008). KIT gene mutations and copy number in melanoma subtypes. Clin Cancer Res.

[20] Larsson KB, Shaw HM, Thompson JF, Harman RC, McCarthy WH (1999). Primary mucosal and glans penis melanomas: the Sidney Melanoma Unit experience. Aust N Z J Surg.

[21] Rohwedder A, Phillips B, Malfetano J, Kredenster D, Carlson JA (2002). Vulvar malignant melanoma associated with human papillomavirus DNA: Report of two cases and review of literature. Am J Dermatopathol.

[22] Lesage C, Barbe C, Le Clainche A, Lesage FX, Bernard P, Grange F (2013). Sex-related location of head and neck melanoma strongly argues for a major role of sun exposure in cars and photoprotection by hair. J Invest Dermatol.

[23] Whiteman DC, Watt P, Purdie DM, Hughes MC, Hayward NK, Green AC (2003). Melanocytic nevi, solar keratoses, and divergent pathways to cutaneous melanoma. J Natl Cancer Inst.

